# Repeat Interventions After Transcatheter Aortic Valve Replacement: Considerations for Lifetime Management—The First Cut Is the Deepest

**DOI:** 10.1016/j.shj.2025.100426

**Published:** 2025-02-15

**Authors:** Sachin S. Goel, Benjamin Z. Galper

**Affiliations:** a Houston Methodist DeBakey Heart and Vascular Center, Houston, Texas, USA; b Mid-Atlantic Permanente Medical Group, McLean, Virginia, USA

Approval of transcatheter aortic valve replacement (TAVR) for the treatment of symptomatic severe aortic stenosis (AS) across all levels of surgical risk has led to an exponential increase in the use of TAVR. The American College of Cardiology/American Heart Association guidelines recommend surgical aortic valve replacement (SAVR) as class I indication for patients <65 years of age, TAVR for patients >80 years of age if anatomy is suitable for TAVR, and individual decision based on patient’s anatomy for TAVR, risk for SAVR, and patient preference for those between aged 65 and 80 years taking into account patient’s expected longevity and durability of TAVR.^1^ The annual TAVR volume has far exceeded the annual isolated SAVR volume for the last several years in the United States. It is important to remember that TAVR vs. SAVR randomized trial results strictly apply to the populations tested and not in general to those excluded from those trials, namely patients with bicuspid aortic valve, severe unrevascularized coronary artery disease, excessive left ventricular outflow tract calcification, and those with a need for concomitant procedures.^2^ The PARTNER low-risk trial had a mean age of 72 years, and the Evolut low-risk trial had a mean age of 73 years, with <7% and <6% of patients aged <65 years in these trials, respectively.^2^ Despite this, we have seen a marked rise in TAVR for the treatment of AS in the United States in patients <65 years of age without long-term durability data on TAVR.^3^ The increase in TAVR in younger populations is likely related to patient-related factors including patient preference, as well as an overzealous adoption of a less invasive strategy, which leads to shorter hospital stays, less resource utilization, and more rapid patient recovery, with the hope that when the transcatheter heart valve degenerates, the patient can undergo redo TAVR if anatomy is suitable or TAVR explant and SAVR.

Not surprisingly, given that many younger low-risk patients are likely to outlive their first aortic valve replacement, some patients with TAVR who have transcatheter heart valve degeneration and bioprosthetic valve failure have returned for consideration of repeat intervention. Data are emerging on the outcomes of both treatment options in this scenario, namely TAVR explant with SAVR and redo TAVR.^4^, ^5^, ^6^, ^7^, ^8^, ^9^, ^10^, ^11^, ^12^, ^13^, ^14^ We have summarized the published outcome data for in-hospital mortality, 30-day, and 1-year mortality following repeat interventions after TAVR failure in Table 1. It is important to note that mortality following TAVR explant with SAVR has been high across various registries, with in-hospital mortality ranging from 11% to 20% and 1-year mortality ranging from 20% to 34%. The outcomes with redo TAVR appear to be much more favorable, with in-hospital mortality ranging from 0% to 2.3% and 1-year mortality ranging from 13% to 21%. The high mortality after TAVR explant is multifactorial and related to high baseline surgical risk with comorbidities, infective endocarditis as an indication for surgery, the need for concomitant procedures such as replacement of the aortic root and ascending aorta, mitral valve surgery, and potentially technical challenge of explanting the TAVR valve given lack of standardization and low volume of such procedures.

**Table 1:** Published data on in-hospital, 30-day, and 1-year mortality for repeat interventions after TAVR

Study	In-hospital mortality	30-d mortality	1-y mortality
Redo TAVR			
Tang G (EXPLANT TO REDO TAVR Registry)^4^	2.3%	3.4%	15.4%
Landes (Redo TAVR registry)^5^	0	2.8%	13.5%
Fukuhara et al.^6^		2%	
Percy et al.^7^		6.2%	21%
Makkar R et al.^8^	0.6%	4.7%	17.5%
Landes et al.^9^		5%	17.4%
TAVR Explant and SAVR			
Tang G (EXPLANT TO REDO TAVR Registry)^4^	11.6%	13.6%	32.4%
Bapat V (EXPLANT TAVR Registry)^10^		13.1%	28.5%
Hawkins et al.^11^	11.3%		
Jawitz et al.^12^	17.1%		
Hirji et al.^13^		13.2%	22.9%
Fukuhara et al.^6^		15%	
Percy et al.^7^		12.3%	20.8%
Brescia et al.^14^	20%	33%	

Abbreviations: TAVR, transcatheter aortic valve replacement; SAVR, surgical aortic valve replacement.

In the current issue of the journal, Mendez-Hirata and colleagues present a single-center experience with repeat interventions after TAVR.^15^ The study included 31 patients; 25 underwent redo TAVR (TAV-in-TAV), and 6 patients underwent TAV explant and SAVR. Most explants were performed for infective endocarditis. The total number of patients considered for repeat intervention after TAVR but deemed unsuitable due to clinical or anatomical reasons is not available. It is somewhat reassuring to see that only 31 out of 1314 (2.3%) patients who had TAVR at the authors’ institution underwent repeat intervention. Median time was 5 years for redo TAVR and 2.6 years for TAVR explant and SAVR. Mechanism of failure for TAV-in-TAV was structural valve deterioration in 60% and nonstructural valve deterioration in 40% (the most common cause was paravalvular leak in 90%). In the TAVR explant group, 5 out of 6 patients had concomitant surgical procedures (commando, root replacement, mitral valve replacement, aortoplasty/root enlargement). For redo TAVR, the most common scenario was balloon-expandable valve (BEV) in BEV (40%), followed by BEV in self-expandable valve (24%). Success rate of redo TAVR was 88%, with 2 in-hospital deaths, 1 stroke, and 3 patients requiring new permanent pacemakers. In the redo TAVR explant group, there was no in-hospital mortality and 1 stroke.

While this small series had no in-hospital mortality after TAVR explant and SAVR, it is important to note that larger multicenter series, summarized in Table 1, have shown a significant risk of in-hospital mortality after TAVR explant. This underscores the importance of choosing the first intervention very carefully, especially in a younger patient with life expectancy greater than that of a bioprosthetic valve. This concept, referred to as lifetime management of AS, should be discussed with every patient by the heart team, particularly for those <70 to 75 years of age. Feasibility of redo TAVR depends largely on anatomy, particularly coronary clearance. While redo TAVR appears to be safe, the denominator, that is, the number of patients screened but declined for redo TAVR due to anatomic reasons, is not available from this study or from the larger Society of Thoracic Surgeons/American College of Cardiology Transcatheter Valve Therapy registry data.^8^ Computed Tomography-based simulation studies show that redo TAVR may not be feasible in a substantial number of patients depending on size and type of index TAVR and patient anatomy. In addition, coronary access for angiography and percutaneous coronary interventions may become very challenging after redo TAVR in some patients. The techniques for TAVR explant and SAVR are being refined, and it is anticipated that the most common valve surgery that will be performed by cardiac surgeons soon may be TAVR explant. Until we have long-term TAVR durability data, younger patients, who are typically low surgical risk, should consider SAVR as the first intervention so that they can safely have valve-in-valve TAVR when the surgical bioprosthesis degenerates. The decision for the first intervention should not be solely based on avoiding an operation. The first cut is indeed the deepest!

## Funding

The authors have no funding to report.

## Disclosure Statement

S. Goel is a consultant for Medtronic, Abbott, Boston Scientific, and JC Medical. B. Z. Galper receives institutional research grant funding from Edwards Lifesciences.
